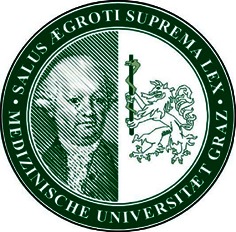# Clinical–Pathological Conference Series from the Medical University of Graz: Case No 154: 32–year–old computer software engineer with nodular mass in the liver

**DOI:** 10.1007/s00508-016-0965-1

**Published:** 2016-02-26

**Authors:** Elisabeth Fabian, Bernhard Haas, Patrizia Kump, Rainer Lipp, Peter Kornprat, Andre Lutfi, Emina Talakic, Michael Fuchsjäger, Walter Spindelboeck, Carolin Lackner, Gernot Zollner, Guenter J. Krejs

**Affiliations:** 1 Division of Gastroenterology and Hepatology, Department of Internal Medicine III, Medical University of Vienna, Vienna, Austria; 2 Division of Gastroenterology and Infectious Diseases, Department of Internal Medicine, Landeskrankenhaus West, Graz, Graz, Austria; 3 Division of Gastroenterology and Hepatology, Department of Internal Medicine, Medical University of Graz, Auenbruggerplatz 15, 8036 Graz, Austria; 4 Division of Nuclear Medicine, Department of Radiology, Medical University of Graz, Graz, Austria; 5 Department of Surgery, Medical University of Graz, Graz, Austria; 6 Department of Radiology, Landeskrankenhaus West, Graz, Graz, Austria; 7 Division of General Radiology, Department of Radiology, Medical University of Graz, Graz, Austria; 8 Department of Pathology, Medical University of Graz, Graz, Austria

**Keywords:** Flush, Neuroendocrine tumor, Liver resection, Somatostatin analog, Portal vein embolization, Carcinoid

## Presentation of case

### Dr. W. Spindelboeck:

Due to episodic epigastric pain this 32-year-old woman had undergone computed tomography (CT) 20 months previously. Contrast enhanced CT showed a hypodense lesion between liver segments IV and VIII with a diameter of 4 cm and inhomogenous early enhancement suggesting hemangioma. Eight more lesions (diameter up to 1 cm) that were only visible in the early arterial phase were found in segments VI and III. Magnetic resonance imaging (MRI) 13 and 8 months before admission showed slight progression (from a diameter of 4.0 to 4.6 cm) of the lesion in segment IV. At that time, the lesion appeared to be lobulated with a central hyperintense scar and arterial enhancement, primarily compatible with “atypical” focal nodular hyperplasia (FNH). Except for occasional abdominal pain and a 10-year history of histamine intolerance, the patient was free of symptoms. She had taken thyroid replacement therapy (Euthyrox® 100 µg per day) for years; she had no previous surgery, had never received a blood transfusion, and was not vaccinated against hepatitis A or B; she neither smoked nor drank and her family history was unremarkable. The patient is a single parent of a healthy 10-year-old boy. Physical examination was unremarkable; she weighed 56 kg and her height was 174 cm. Except for lactate dehydrogenase (LDH:296 U/l, normal 120–240 U/l) routine laboratory tests were negative. A panel of antibodies to detect autoimmune and collagen vascular diseases was negative; thyroid stimulating hormone (TSH) was 1.3 µU/ml (normal 0.1–4.0 µU/l).

During a follow-up exam in the outpatient liver clinic, she showed a facial flush that lasted for 2 min, although the situation was not psychologically upsetting. She said facial flushes are part of her histamine intolerance. A diagnostic test was performed, and she was admitted to the hospital for further management.

### Dr. M. Fuchsjäger:

**Fig. 1:**
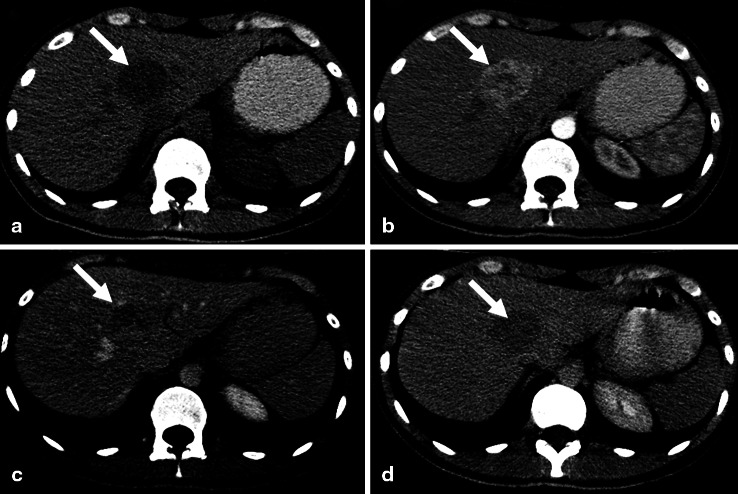
Multidetector CT, axial images. 4.4 × 3.3 cm, well defined, heterogenous, and hypodense lesion in liver segments VIII and IV upon pre-contrast scan (a); lesion demonstrated significant enhancement in arterial (b) and less enhancement in portal venous phases (**c**); no enhancement of the irregular area within the lesion, and definite contrast washout pattern was present in delayed phase (**d**)

**Fig. 2:**
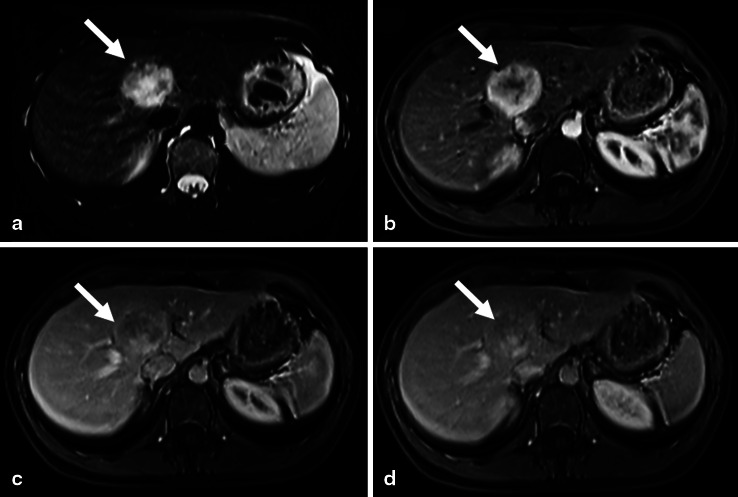
MRI: T2W image demonstrates a large, well-circumscribed hyperintense mass (**a**). Post-contrast T1W images showed highly heterogenous arterial enhancement, the central hyperintense area probably represents necrosis (**b**). In portal venous (**c**) and delayed phases (**d**), the lesion is iso- to hypointense to liver parenchyma with the central portion remaining almost nonenhanced.

Abdominal multidetector CT revealed a 4.0 × 3.3 cm, well circumscribed, heterogenous, hypodense lesion in liver segments VIII/IV with significant contrast enhancement during the arterial and portal venous phases and with contrast washout during delayed phases (Fig. 1). Eight more lesions (diameter up to 1 cm), only visible in the early arterial phase, were found in segments VI and III. MRI studies of the liver 7 and 12 months later both showed multiple, well-circumscribed, heterogenous, hypointense hepatic mass lesions with significant contrast enhancement (Fig. 2). Between the 13th and 8th months before admission, the target lesion increased slightly in size, from 4.0 to 4.6 cm.

Taken together, the MRI studies showed hypervascular lesions in both lobes of the liver compatible with several diagnoses. The hyperintensity of the larger lesion and the early arterial contrast enhancement also seen in the smaller lesions are typical for hemangiomas. Moreover, upon MRI hemangiomas show a quite homogenous portal venous contrast enhancement due to accumulation of contrast medium during the examination. So in this case, MRI suggests a hemangioma for several reasons. On the other hand, the central hyperintense scar found in one of the lesions could be characteristic for FNH; however, the MRI findings do not clearly allow a definite diagnosis.

## Differential diagnosis

### Dr. B. Haas:

The patient under discussion is a 32-year-old woman with nodules in the liver. Except for occasional abdominal pain and histamine intolerance, she is asymptomatic. Twenty months earlier, CT of the liver showed what was first thought to be a hemangioma and later interpreted as an “atypical” FNH. Further MRIs revealed progression of the lesion (from a diameter of 4.0 to 4.6 cm) and identified eight additional lesions (diameter up to 1 cm) in the liver. Facial flushes were thought by the patient to be due to histamine intolerance diagnosed 10 years before. Physical examination did not show any abnormality and, except for LDH, routine laboratory tests were within normal limits.

As the patient is a native of Austria, hepatocellular carcinoma (HCC) in a noncirrhotic liver due to chronic hepatitis B is unlikely. Since hepatitis serology is routinely tested during pregnancy in this country, an infection would have been revealed much earlier when the patient had been pregnant. It is unclear whether the patient had consumed foods such as wild berries or raw vegetables and so might have been infected with *echinococcus*; however, the MRI lesions are not typical for echinococcal cysts [1]. An infection with *Bartonella henselae* due to contact with cats and a resulting bacillary peliosis hepatis could be considered, but this disease predominantly occurs in immunosuppressed patients, is associated with fever, and runs a much shorter course. As to the episodic abdominal pain, a regular recurrence of symptoms could parallel the menstrual cycle, but endometriosis would not really explain the liver lesions. Since the panel of antibodies for autoimmune and collagen vascular diseases was negative, an autoimmune disease can alsobe ruled out. The patient said she was histamine intolerant, but unfortunately no further information was available as to how this diagnosis was established 10 years earlier (genetic variant? activity of diaminooxidase?). FNH could still be considered as a possible diagnosis, but it would not have required prompt admission to the hospital. The assumed diagnosis of a liver hemangioma based on the CT scan, which showed a hypodense lesion with inhomogenous early arterial contrast enhancement, appears unlikely since the lesion had progressed in size.

For my differential diagnosis, I have to address the facial flush presumed to be due to histamine intolerance. Flush symptoms can be caused by enhanced release of vasoactive substances such as serotonin and bradykinin, mostly due to a gastroenteropancreatic neuroendocrine tumor (GEP-NET). Frequently, flush symptoms in GEP-NETs occur in the setting of liver metastases. This is because most of the bioactive substances that are released by a GEP-NET may be metabolized by the liver on first pass from the splanchnic area and, more importantly, liver metastases provide for more tissue for production and release of bioactive substances into the circulation. Laboratory tests showed elevated serum LDH, with an increased ratio of LDH/AST, a possible marker for the presence of liver metastases. An important diagnostic test that could suggest a GEP-NET is the analysis of serum chromogranin A. Chromogranin A is a protein found in the secretory granules of neuroendocrine cells, and its concentration correlates with tumor mass [2]. Further diagnostic steps include analysis of 5-hydroxyindol acetic acid (5-HIAA) in a 24-h urine sample. Endoscopic investigation including capsule endoscopy of the small bowel, abdominal sonography, and contrast-enhanced CT, PET, and radionuclear imaging such as ^99m^technetium octreotid scintigraphy and ^68^gallium-DOTATATE (= DOTADOC) PET-CT are parts of the further workup.

## Dr. B. Haas’s diagnosis

GEP-NET with liver metastases; clinically “carcinoid syndrome.”

## Discussion of diagnosis

### Dr. W. Spindelboeck:

This patient does indeed have a gut neuroendocrine tumor (NET). Further history revealed that flush symptoms as noticed in the outpatient liver clinic occur 10 to 50 times per day. The patient complained of bloating but denied diarrhea. The following laboratory results were obtained: serum chromogranin A 1061 ng/ml (normal 0–99 ng/ml), serum serotonin 2063 ng/ml (normal 80–450 ng/ml), and urinary 5-HIAA 118 mg/24h (normal 6–10 mg/24h). For further staging, MRI of the small intestine and the liver was performed.

### Dr. G. J. Krejs:

Just a short remark—the patient was admitted immediately so that somatostatin analog therapy could begin without delay.

Some epidemiological facts: Although previously regarded as rare, GEP-NETs represent the second most common digestive malignancy after adenocarcinomas [3, 4]. Based on data of the Surveillance, Epidemiology and End Results (SEER) program of the National Cancer Institute including 29,664 patients, the incidence is estimated to be 3.65/100,000 persons per year [5]. In Austria, Dr. Niederle found a similar incidence [6]. The incidence of GEP-NET has increased in recent decades; the expanding use of sophisticated imaging studies is believed to play a role in this development, but there seems to be a true increase. GEP-NETs mostly occur in the small intestine (31 %), followed by rectum (26 %), colon (18 %), pancreas (12 %), and appendix (6 %) [7]. The presence of liver metastases depends on the site of the primary tumor, tumor extent (T-stage), histological differentiation, and proliferative activity (grading; G1-G3). Pancreas, right hemicolon, and small intestine are the most frequent primary tumor sites presenting with distant metastases upon initial diagnosis. Some data show that 80–90 % of patients with small intestinal neuroendocrine neoplasia also have liver metastases [8]. In patients with “carcinoid syndrome,” distant metastases are regularly observed, and they are found more frequently in patients with poorly differentiated endocrine carcinoma (NEC G3) than in those with well-differentiated NET G1-G2 [8]. Metastases in NET patients can only be assessed with sensitive imaging techniques. In addition to MRI, radionuclear imaging was also performed in the discussed patient, and Dr. Lipp will show the results.

### Dr. R. Lipp:

NET cells express somatostatin receptors (SSR) which are the target for radionuclear tracers during somatostatin-receptor scintigraphy (SSRS). For SSRS, a gamma emitter (single-photon emission CT, SPECT) such as ^111^In-DTPA-octreotide (OctreoScan™) and ^99m^Tc-tektrotyde^®^, or a positron emitter (positron emission tomography, PET) such as ^68^Ga-octreotide, ^68^Ga-DOTA-TOC, and ^64^Cu-DOTA-TATE is used.

^18^Fluro-DOPA PET/CT is another radionuclear method to assess the metabolic activity of GEP-NET cells independent of the SSR status. In NET cells the activity of DOPA-decarboxylase that decarboxylates the aminprecursor DOPA to a biogenic amin is increased [9–11]. Depending on the degree of differentiation, GEP-NET cells take up and metabolize ^18^fluro-DOPA differently. In one third of GEP-NET patients this investigation provides pivotal and therapeutically important information that cannot be obtained by other morphologic and functional imaging methods [12].

**Fig. 3:**
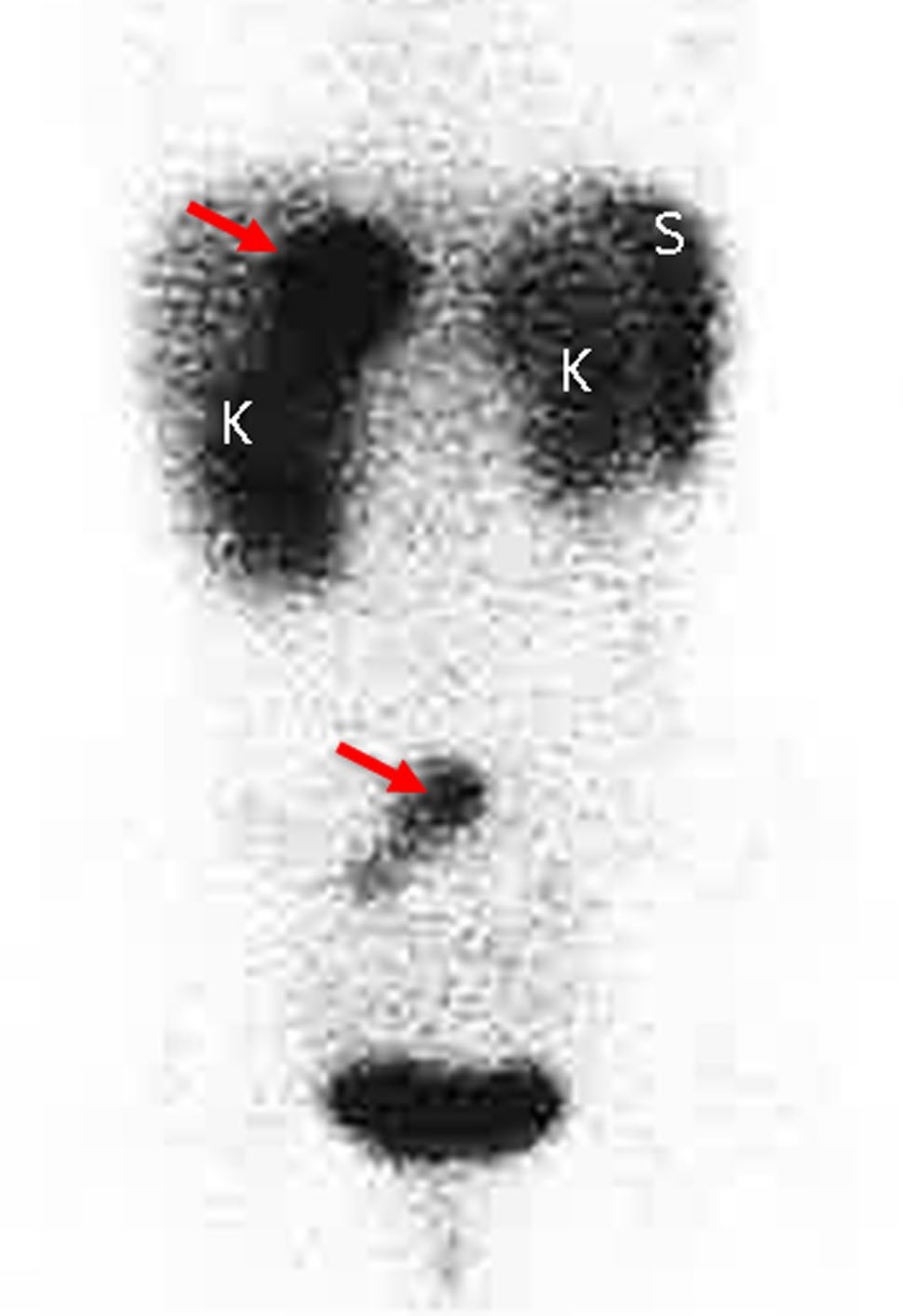
Somatostatin receptor imaging with ^111^In-pentetreotide (OctreoScan™) showing somatostatin receptor expression of the liver metastasis in liver segment IV and additionally in mesenteric lymph nodes (*red arrows*). Normal enhancement is seen in spleen (S), kidneys (K), and urinary bladder (U)

**Fig. 4:**
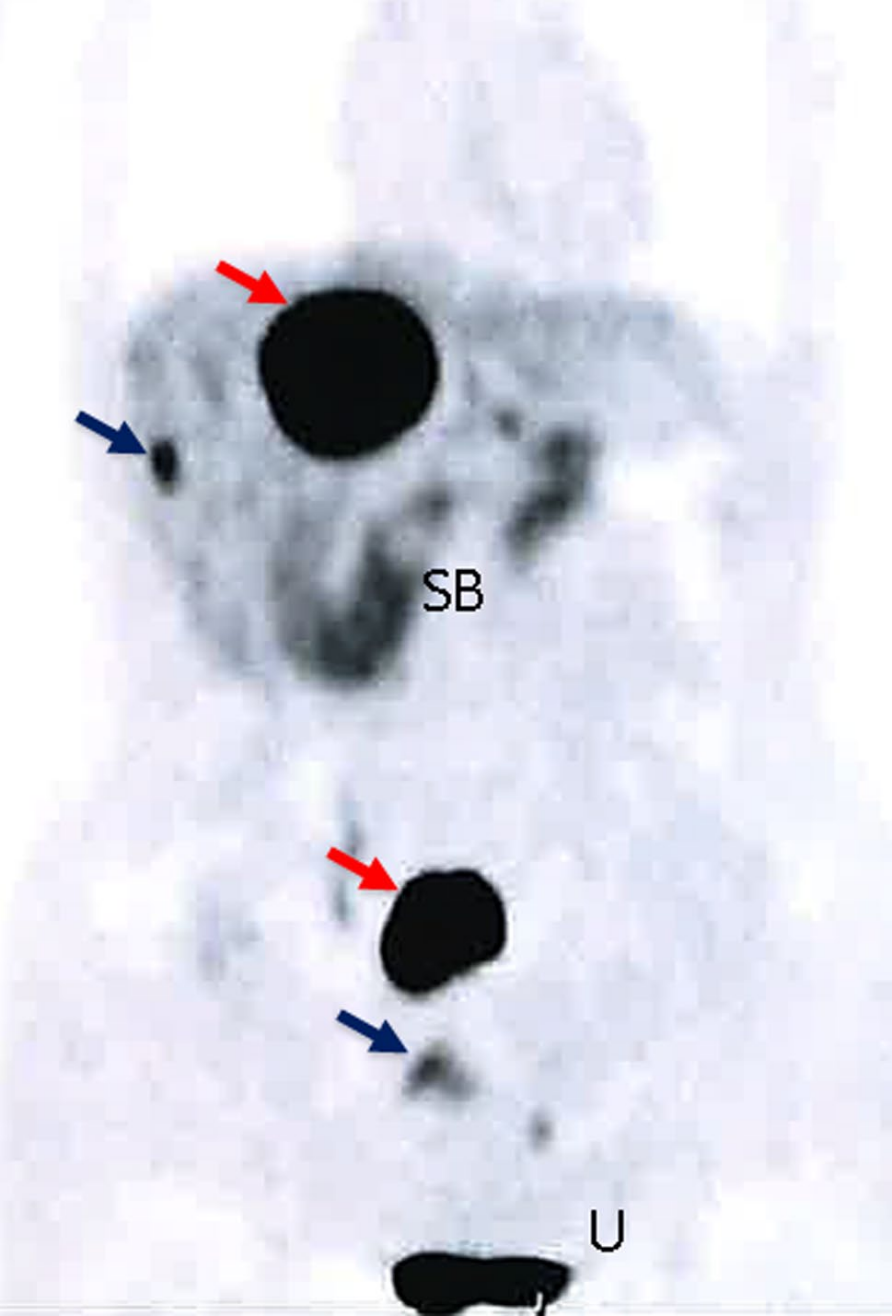
^18^Fluro-DOPA PET showing increased DOPA metabolism in neuroendocrine metastases in the liver and mesenteric lymph nodes (*red arrows*). Note additional metastatic sites in the right lobe of the liver and pelvis not shown in somatostatin receptor imaging (*blue arrows*). Normal enhancement due to biliary and urinary excretion of the marker is seen in the upper small bowel (SB) and urinary bladder (U)

This patient underwent SSRS (Fig. 3) and ^18^fluro-DOPA PET/CT (Fig. 4) to confirm the final diagnosis and her regular follow-up includes these studies.

### Dr. M. Fuchsjäger:

Radiologic imaging currently lacks specificity for GEP-NETs, which are often mistaken for more common lesions. Due to their highly variable appearance, NET liver metastases may first be taken for benign lesions such as adenoma or hemangioma or confused with another hepatic malignancy such as HCC or cholangiocarcinoma. Since these imaging findings may overlap with other liver neoplasms, diagnosis of liver metastases from NETs still rests primarily on pathological analysis and immunochemistry of biopsy and/or surgical specimens [13].

Liver metastases seen on CT images most frequently are less attenuated than surrounding liver parenchyma on pre-contrast images but strongly enhance post-contrast, mimicking hemangioma. Metastases of NETs may be difficult to identify and delineate on CT as they may be isodense with the liver on portal venous phase imaging. In some cases a lesion may be seen only on one of the three phases (pre-contrast, arterial phase, and portal venous phase) [14, 15].

MRI has a higher sensitivity for identifying the primary tumor. Tumors usually have low signal intensity on T1-weighted sequences (75 %) and high signal intensity on T2-weighted sequences (94 %), being hypervascular on arterial post-gadolinium images: 15 % of liver metastases were only seen on the immediate post-gadolinium images. The tumors are most conspicuous on fat-suppressed T1-weighted images [15, 16]. Both CT and MRI can be used to stage nodal and distant metastatic disease as part of preoperative planning [17].

### Dr. G. J. Krejs:

After the GEP-NET had been diagnosed the patient was immediately treated with somatostatin analog (SSA) and 3 months later underwent tumor debulking, i.e. resection of the primary tumor and liver metastases. Dr. Kornprat was the surgeon and will explain and comment on the treatment.

### Dr. P. Kornprat:

Surgical resection of liver metastases is known to have a positive effect on overall survival and quality of life due to alleviation of symptoms related to secretion of serotonin or other mediators in functioning tumors [8]. Sarmiento et al. reported that 95 % of patients with specific symptoms at the time of surgery experienced improvement afterwards. According to the European Neuroendocrine Tumor Society (ENETS) Guidelines surgical resection with curative intent remains the gold standard in the treatment of liver metastases, achieving a survival rate of 60–80 % at 5 years. Prerequisites are (1) a tumor classification of NET G1/G2, i.e. well-differentiated, resectable lesions with acceptable morbidity and < 5 % mortality; (2) absence of right ventricular failure; (3) absence of unresectable lymph nodes and extra-abdominal metastases; and (4) absence of diffuse or unresectable peritoneal carcinomatosis [8].

When NETs are associated with endocrine syndromes, debulking surgery is attempted whenever feasible. Debulking procedures include not only resection of the primary tumor, liver metastases, and lymph nodes but also ablative therapies that remove > 90 % of the tumor [8, 18–20]. In patients with “carcinoid syndrome,” perioperative treatment with SSA is indicated to prevent intra- and postoperative carcinoid crisis [21, 22].

A two-step surgical approach was chosen for this patient: First, a 75-cm-long segment of the lower small bowel with adjacent mesenteric nodes was resected. There were no intra- or postoperative complications.

**Fig. 5:**
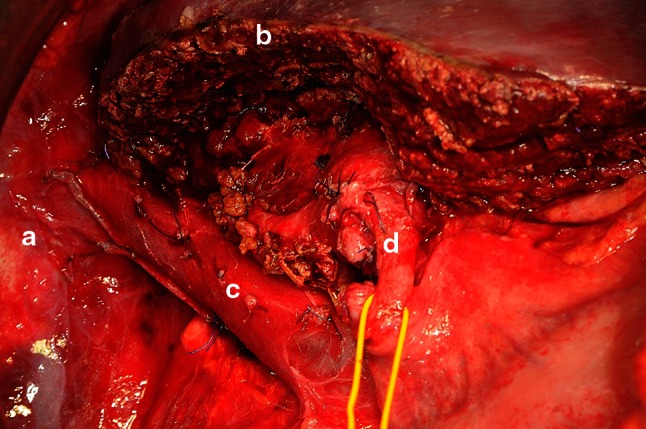
Intraoperative image after completion of the extended hemihepatectomy. (**a**) right diaphragm, (**b**) margin of resection at the remaining left lobe of the liver, (**c**) inferior vena cava, (**d**) common bile duct

Depending on the number and localization of the metastases, up to 65–70 % of the whole liver volume (in patients with normal liver parenchyma) can be removed surgically [23, 24], and large parts of our patient’s liver had to be resected. Since the future parenchymal remnant would have been too small, the right portal vein branches had to be embolized to induce hypertrophy of the left lobe of the liver. This was done 17 days after the first operation and 10 weeks before an extended hemihepatectomy of the right lobe with an atypical resection of segment III and lobus quadratus (Fig. 5) could be carried out. The second operation was also performed without complications.

### Dr. C. Lackner:

Macroscopically, the resected segment of ileum was 75 cm long and contained three tumors of 2, 1.5, and 3 cm in diameter. The tumors were 1.5 and 6 cm apart and were located 20 cm from both, the oral and aboral margin of resection. All three tumors extended beyond the mucosa into the small bowel wall. The serosa was intact. The mesentery showed another mass of 5.0 × 3.5 × 3.0 cm that extended to the resection margin and five lymph nodes with diameters of up to 2 cm.

**Fig. 6:**
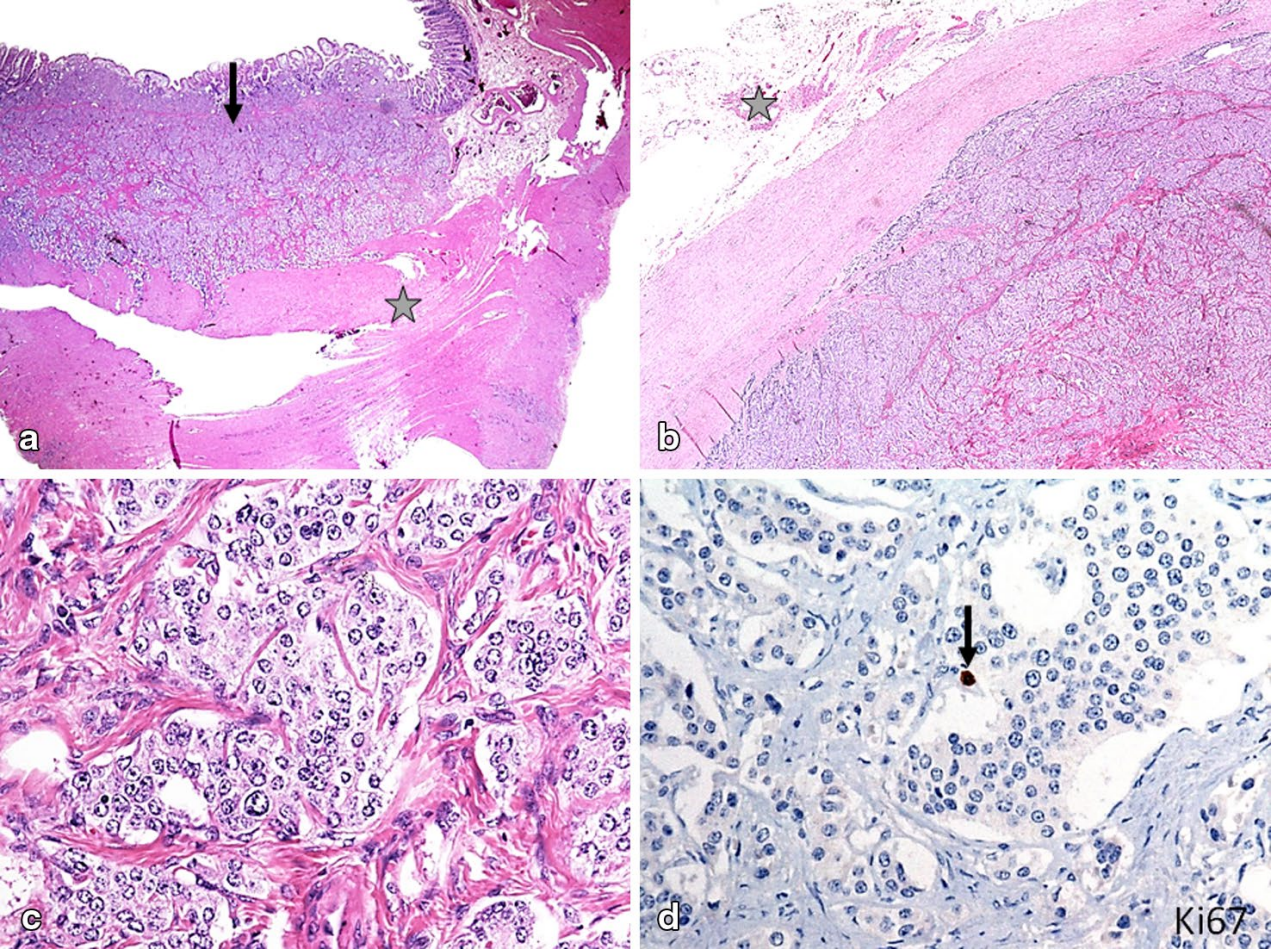
NET of the ileum with metastasis to the mesentery. (**a**) NET (arrow) extending to the muscularis propria (indicated by star) of the ileum (H&E; 20x); (**b**) metastasis to the mesentery (indicated by star), surrounded by fibrous pseudocapsule (H&E; 20x); (**c**) tumor cells with mild cytological atypia and enlarged nuclei with salt-and-pepper chromatin (H&E; 400x); (**d**) very few tumor cell nuclei stain with antibodies against Ki67 (arrow, hematoxyline, 400x)

Microscopically, all three tumors found in the resected ileum and the tumor in the mesentery could be identified morphologically and immunohistochemically as NETs (Fig. 6). They were well demarcated and extended to the subserosa.

Systems of nomenclature reflect differentiation and grading features of NETs (Table 1). Generally, NETs are divided into well-differentiated and poorly differentiated tumors (the latter referred to as neuroendocrine carcinomas: NEC). Differentiation and grade of the tumor are linked; there are, however, subtle differences between the two concepts. “Differentiation” refers to the extent to which the neoplastic cells resemble their non-neoplastic counterparts. Well-differentiated NETs have characteristic organoid arrangements of tumor cells, with nesting, trabecular or gyriform patterns; the cells are relatively uniform with monomorphic nuclei. They produce abundant neurosecretory granules, reflected by the strong immunoexpression of neuroendocrine markers such as chromogranin A. NECs less closely resemble non-neoplastic neuroendocrine cells, and their architecture is less well organized. The tumor cells contain enlarged polymorphic and hyperchromatic nuclei and sparse cytoplasm. They produce fewer neurosecretory granules and neuroendocrine markers. “Grade” refers to the inherent biologic aggressiveness of the tumor. Low-grade NETs are relatively indolent; intermediate-grade tumors show less predictable, moderately aggressive behavior; and high-grade tumors (i.e., NECs) are extremely aggressive [25].

**Table 1:** Systems of nomenclature and grading of GEP-NETs

Grade (ENETS, WHO) [26, 27, 29]		ENETS [26, 27]	WHO 2010 [29]
Low grade	< 2 mitoses/10 HPF and < 3 % Ki67 index	Neuroendocrine tumor, grade 1 (G1)	Neuroendocrine neoplasm, grade 1
Intermediate grade	2–20 mitoses/10 HPF or 3–20 % Ki67 index	Neuroendocrine tumor, grade 2 (G2)	Neuroendocrine neoplasm, grade 2
High grade	> 20 mitoses/10 HPF or > 20 % Ki67 index	Neuroendocrine carcinoma, grade 3 (G3), small cell carcinoma	Neuroendocrine carcinoma, grade 3, small cell carcinoma
		Neuroendocrine carcinoma, grade 3 (G3), large cell neuroendocrine carcinoma	Neuroendocrine carcinoma, grade 3, large cell neuroendocrine carcinoma

*HPF* high-power microscopic fields, *ENETS* European Neuroendocrine Tumor Society, *WHO* World Health Organization

The proliferative rate of a NET provides significant prognostic information and can be assessed as the number of mitoses per unit area of tumor (usually expressed as mitoses per 10 HPF) or as the percentage of neoplastic cells immunolabeling for the proliferation marker Ki67 [26, 27].

In this patient, fewer than two mitoses/10 HPF and a Ki67 index ≤ 2 % were found, corresponding to NET grade 1 (NET G1). The tumor in the mesentery extended to less than 1 mm from the resection margin, so that there was some uncertainty as to whether the tumor had been completely resected. There were neural sheath and venous invasions at multiple sites in the area of the mesenteric root. Moreover, a metastasis was found in one of the five lymph nodes in the resected mesentery. The tumor cells were immunohistochemically positive, with antibodies against chromogranin and synaptophysin. Some of the tumor cells reacted with antibodies against serotonin but not with those against gastrin, adrenocorticotropin, somatostatin, and pancreatic polypeptide.

The liver specimen measured 17.0 × 10.5 × 5.0 cm. A tumor of 5.5 × 4.0 × 4.0 cm extending to the resection margin was found on the cut surface, which on histology proved to represent a liver metastasis of the NET described above.

According to the UICC 2009 [28] the tumor could be classified as G1 pT3(m) N1 M1 (HEP) V1 Pn1, R2.

The prognosis for jejunoileal NETs depends on tumor size and the extent of tumor infiltration, the grade of differentiation, and the presence of metastases. With liver metastases, the 5- and 10-year survival rates amount to 35 and 15 %; without liver metastases the rates increase to 72 and 60 %, respectively [29].

### Dr. G. J. Krejs:

The term NET was based on the hypothesis that the cells of neuroendocrine neoplasms originate from the embryonic neural crest. Based on findings that most of these neoplastic cells resemble cells of endodermal origin, this concept was discarded years ago [25]. However, neoplastic cells possess features of both neural and epithelial cells, and for this reason, the most recent WHO classification of tumors of the digestive system has once again recommended the use of the term “neuroendocrine” [29]. NETs are diverse in their site of origin and clinical behavior, ranging from highly aggressive cancers to low-grade tumors of the small bowel. Symptoms arise from both the tumor burden and the secretion of bioactive hormones by “functioning tumors” as observed in this patient. While high-grade tumors (G3) are often treated with chemotherapy, somatostatin analogs to alleviate the consequences of hormone secretion of “functioning tumors” are the appropriate therapy for low-grade NETs [30, 31]. Recent studies on treatment of pancreatic NETs with sunitinib [32] and everolimus [33] provide evidence for the importance of angiogenesis and the mTOR pathway in the growth of NETs, but there is still a significant unmet need to improve outcomes in this disease. Due to the diagnosis of a low-grade GEP-NET, our patient was treated with somatostatin analog before surgery. Dr. Kump is her attending physician and will report on the therapeutic progress and follow-up of the patient under somatostatin analog therapy.

### Dr. P. Kump:

G protein-coupled receptors (GPRs) in NETs have been studied extensively. Since stimulation or inhibition of such receptors can influence tumor growth, a number of GRPs have been found to be excellent targets for GEP-NET diagnosis and treatment [34]. Somatostatin receptors (SSRs) belong to the GPR family, are expressed on the cell membranes of various tumors including GEP-NET, and bind somatostatin and its therapeutic analogs (octreotide and lanreotide) [35]. Targeting SSRs with somatostatin analogs (SSAs) can block the secretion of biologically active substances from the tumor cells [36–38] and may inhibit cell growth and induce apoptosis [39]. Some tumors, however, are resistant to SSAs and it is not known whether the defect lies in the activation of the SSR or downstream signaling events [31]. After our patient received a SSA (lanreotide 120 mg/month) preoperatively, there was a major decrease in chromogranin A levels from 1068 to 180 ng/ml (upper limit of normal 99 ng/ml) with a reduction in the frequency of flushes from > 20 per day to 2–3 per day. To prevent a carcinoid crisis, SSA therapy should always be started preoperatively and should be continued throughout the surgery.

After resection of the primary tumor, subsequent extended hemihepatectomy and continued lanreotide treatment, chromogranin A levels further decreased to 71 ng/ml. Due to vertigo and hypotension as side effects, the dosage of lanreotide had to be reduced from 120 mg/month to 90 mg/month. One year after surgery, staging revealed micrometastases in the remaining liver on PET scan, but they were not detectable by MRI; the disease state so was classified as “stable.” The patient has no more flushes, and urinary 5-HIAA is not elevated. According to the CLARINET (*A Randomized Double-Blind Placebo-Controlled Study of Lanreotide Antiproliferative Response in Patients with Gastroenteropancreatic Neuroendocrine Tumors*) study, lanreotide (Somatuline Autogel®) significantly prolongs the progression-free survival (PFS) of patients with GEP-NETs (*p* = 0.0002; hazard ratio 0.47; 95 % CI: 0.30–0.73). The antiproliferative effect of lanreotide is statistically significant for patients with midgut NET and clinically relevant for those with pancreatic NET. As treatment with lanreotide also prolongs the PFS of GEP-NET patients with high-grade tumors (G2, Ki67 3–10 %) and higher hepatic tumor burden (> 25 %) [40], treatment with SSA is currently the best therapeutic option for our patient to prolong PFS. At the time of submission of this manuscript she had already been followed for 4 years and is doing well.

### Dr. G. J. Krejs:

Which further or future therapeutic options could be considered for this patient? Could liver transplantation be an option?

### Dr. P. Kump:

According to the ENETS Guidelines [8], SSA therapy and tumor debulking comprise the first therapeutic approach for patients with a functioning NET. With further progression of the tumor and positive SSRs, radioligand therapy or treatment with mTOR inhibitors such as everolimus and sunitinib should be the next therapeutic step. In patients with G1 NETs, chemotherapy is not generally useful because sensitivity to this therapy depends on the proliferation index of the tumor and increases with the NET grading [41]. Currently, there is no clear cut-off value for Ki67 for recommendation of chemotherapy, but depending on tumor-related local symptoms, high liver tumor burden, or tumor progression, chemotherapy should be considered individually even for NETs with a lower proliferation index.

Liver transplantation may be a therapeutic option for patients with functioning NETs refractory to medical therapy that can lead to life-threatening hormonal disturbances, or for patients with nonfunctioning tumors with diffuse unresectable liver metastases refractory to all other possible treatments [8]. Consideration of liver transplantation should be based on the following criteria: (1) well-differentiated NET (G1, G2), Ki67 < 10 %; (2) resected primary tumor; (3) absence of extrahepatic metastases determined by PET/CT; and (4) free of progression for 2–3 years. Patient age of less than 50 years is also a favorable prognostic parameter. Since only a very small percentage of patients with liver transplantation are still tumor-free after 5 years, palliation is the realistic aim of transplantation. Long-term disease-free survival, i.e. liver transplantation with intent to cure, remains the exception [8]. We will have to wait and see whether our patient becomes a candidate for liver transplantation.

### Dr. G. J. Krejs:

This case clearly shows that initial imaging studies can sometimes be misleading and that facial flush is not necessarily due to histamine intolerance. In a recent case report, a patient had been followed for a serous cyst in the liver for many years before frequent flushing developed and a NET was diagnosed [42]. On the other hand, not every flush originates from a NET (Table 2). Now some final remarks from the discussant.

**Table 2:** Causes of a flush

Psychological
Alcohol
Menopause
Basophilic granulocytic leukemia
Systemic mastocytosis
“Carcinoid syndrome”
VIPoma (Verner-Morrison syndrome)
Medullary carcinoma of thyroid
Monosodium glutamate (Chinese restaurant syndrome)

### Dr. B. Haas:

Patients should always be examined very carefully even if the primary assessment suggests such harmless conditions as hemangioma of the liver or histamine intolerance. A history of flushes should lead the physician to rule out a dangerous cause of flush, such as malignant “carcinoid syndrome” (metastatic NET).

## Final diagnosis

Three neuroendocrine tumors of the ileum (G1, “carcinoids”) with metastases to the mesenteric lymph nodes and the liver.